# Supplementary material for: HDAC3 Activity is Essential for Human Leukemic Cell Growth and the Expression of β-catenin, MYC, and WT1
Source: Cancers (Basel). 2019 Sep 26;11(10):1436. doi: 10.3390/cancers11101436 (PMC6826998; doi:10.3390/cancers11101436)
Supplement: Supplementary file 1 [file cancers-11-01436-s001.pdf]

Supplementary information

Supporting Information is available for this paper.

Supplement 1: Examples of dot plots collected by flow cytometry of MV4-11 and HEL cells

Dot plots illustrating the responses of MV4-11 and HEL cells to 30 nM LBH589 and 1 µM Marbostat-100 (M-100) after 24 h. The x-axis are annexin-V-positive cells and the y-axis are PI-positive cells. Early apoptotic cells are annexin-V-positive and late apoptotic cells are annexin-V/PI-positive.

Supplement 2: Apoptosis induction in MV4-11 and HEL after LBH589 treatment

Apoptosis of MV4-11 and HEL cells was measured by flow cytometry after annexin-V-APC/7AAD-staining. Cells were exposed to 10 nM or 30 nM LBH589 for 48 h. Data shown are the mean values + SD (n=3). The pro-apoptotic effects were significant with $p<0.0001$ for both cell lines (t-test).

Supplement 3: Effects of HDACi and z-VAD-FMK on β-catenin status, caspase-3 cleavage, and the HDACi-induced accumulation of ɣH2AX

MV4-11 cells were pretreated with 40 µM of the pan-caspase inhibitor z-VAD-FMK for 1 h followed by stimulation with 30 nM LBH589, 5 µM MS-275, or 10 nM FK228 for 24 h. Whole cell extracts were blotted for β-catenin, cleaved (cl.) caspase-3, and γH2AX; GAPDH as loading control (n=2).

Supplement 4: Flow cytometry assessing effects of LBH589 on KG1 cells

KG1 cells were treated with increasing concentrations of LBH589 (10 nM, 30 nM, and 50 nM) for 48 h. Cells were stained with annexin-V-FITC/PI and analyzed by flow cytometry; mean + SD, n=3. These effects were significant for early apoptosis (**** $p < 0.0001$; two-way ANOVA). On the right, we show exemplary dot plots.

Supplement 5: LBH589 is effective in FFM12 cells

AML-LTCs FFM12 were exposed to DMSO or 10-30 nM LBH589 for 25 h. Immunoblot was done for MYC, acetylated (ac) histone H3, and β-actin as loading control.

Supplement 6: Examples of dot plots collected by flow cytometry of RGFP966-treated MV4-11 cells

Dot plots illustrating the responses of MV4-11 to 1-10 µM RGFP966 after 24 h.

Supplement 7: HU decreases β-catenin and MYC

The human AML cell line MV4-11 was treated with increasing doses of hydroxyurea (HU) or 30 nM LBH589 for 24 h. Indicated proteins were detected via Western blot. GAPDH serves as loading control (n=2).

Supplement 8: Full scans of Western blot data

Beyer_Romanski_Supplement 1

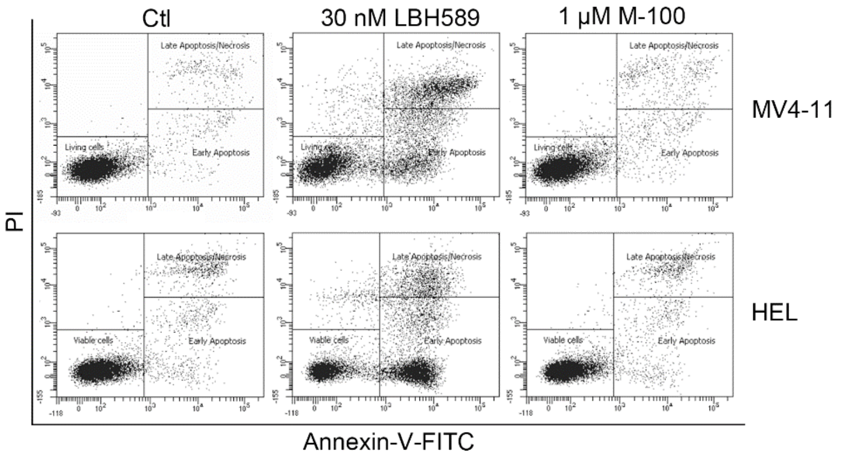

Beyer_Romanski_Supplement 2

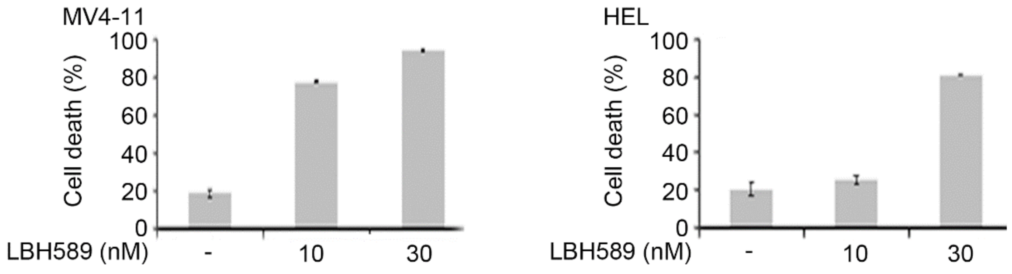

Beyer_Romanski_Supplement 3

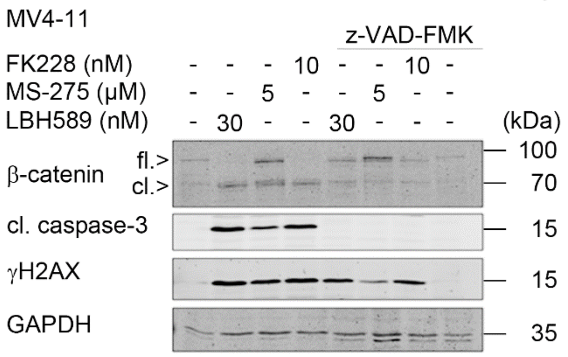

Beyer_Romanski_Supplement 4

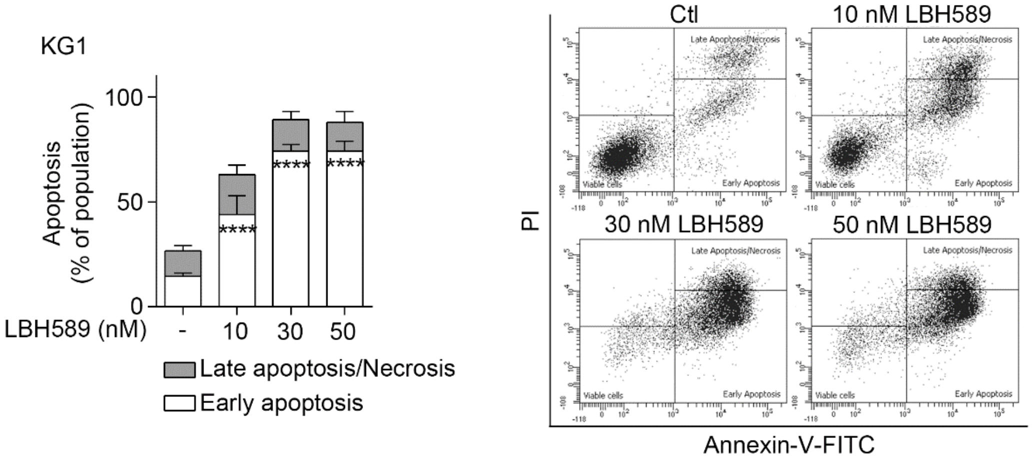

Beyer_Romanski_Supplement 5

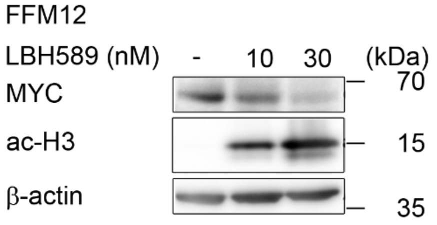

Beyer_Romanski_Supplement 6

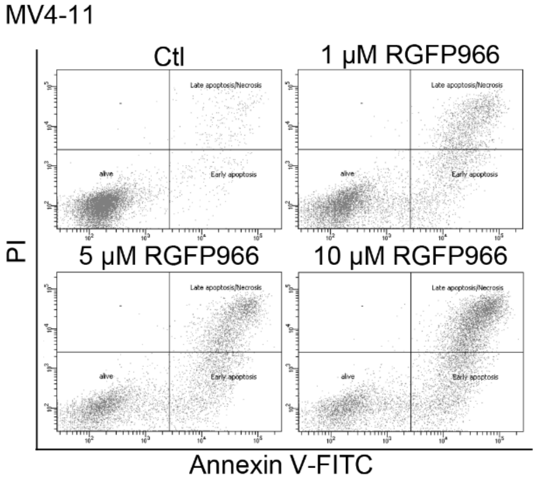

Beyer_Romanski_Supplement 7

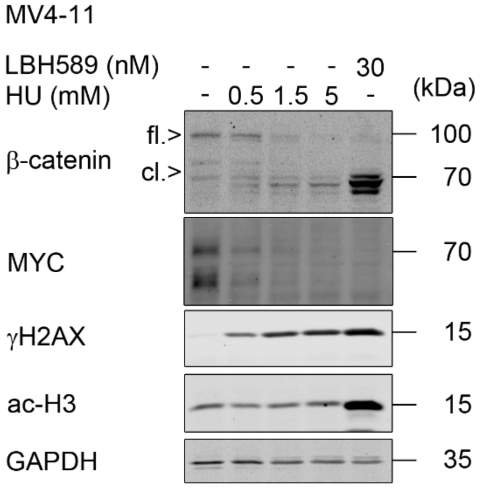

Original blots corresponding to Figure 1C

MV4-11

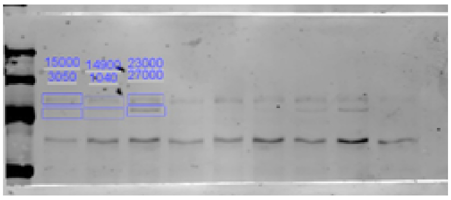

PARP1

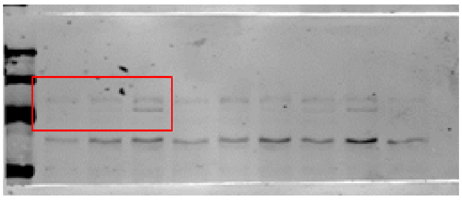

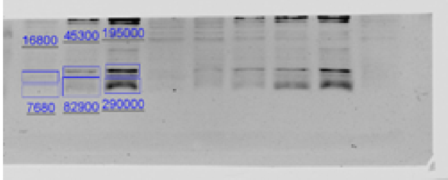

cl. caspase-3

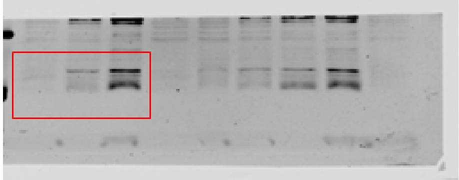

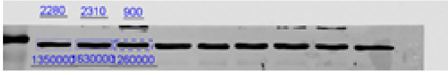

HSP90

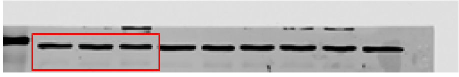

HEL

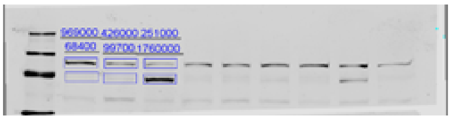

PARP1

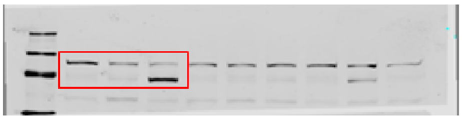

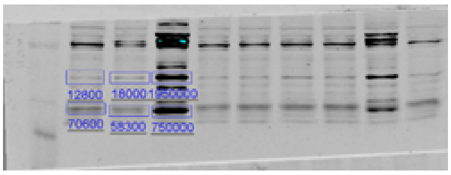

cl. caspase-3

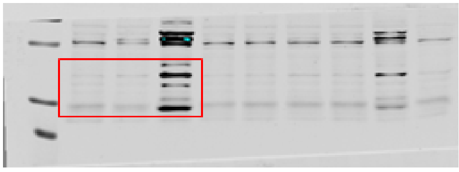

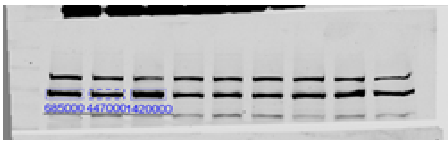

HSP90

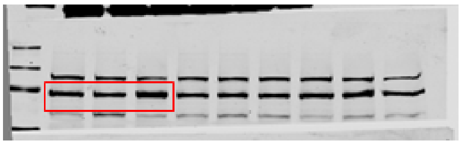

MV4-11	HSP90		PARP1. fl		PARP1. cl		cl. caspase-3		cl. caspase-3	
	Signal	Ratio	Signal	Ratio	Signal	Ratio	Signal	Ratio	Signal	Ratio
10 nM LBH589	1260000	0.9333333	23000	0.017037	27000	0.02	195000	0.1444444	290000	0.2148148
30 nM LBH589	1630000	1.2074074	14900	0.011037	1040	0.0007704	45300	0.0335556	82900	0.0614074
Ctl.	1350000	1	15000	0.0111111	3050	0.0022593	16800	0.0124444	7680	0.0056889

HEL	HSP90		PARP1. fl		PARP1. cl		cl. caspase-3 (i)		cl. caspase-3 (ii)	
	Signal	Ratio	Signal	Ratio	Signal	Ratio	Signal	Ratio	Signal	Ratio
10 nM LBH589	1420000	2.0729927	251000	0.3664234	1760000	2.5693431	1950000	2.8467153	750000	1.0948905
30 nM LBH589	447000	0.6525547	426000	0.6218978	99700	0.1455474	18000	0.0262774	58300	0.0851095
Ctl.	685000	1	969000	1.4145985	68400	0.099854	12800	0.0186861	70600	0.1030657

MV4-11 - Original blots corresponding to Figure 2A

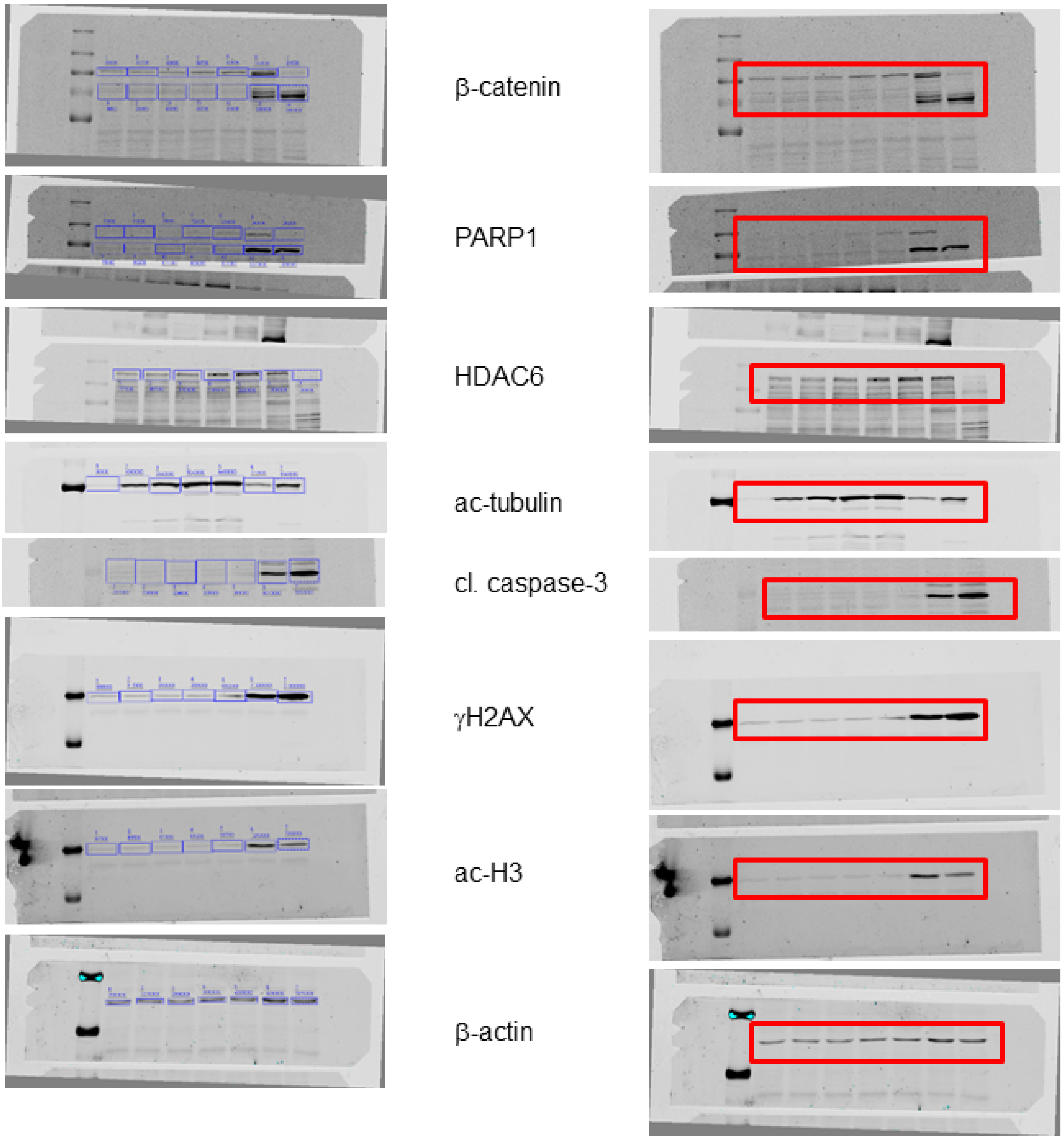

	catenin fl.		catenin cl.		PARP1 fl.		PARP1 cl.		HDAC6		ac-tubulin	
	Signal	Ratio	Signal	Ratio	Signal	Ratio	Signal	Ratio	Signal	Ratio	Signal	Ratio
Ctl	34563,25	1	9464,125	1	76934	1	75320,5	1	776684	1	95974	1
50 nM M-100	37193,844	1,07611	26932,875	2,845786061	71489	0,9292251	93185	1,2372	887278	1,1424	1602155	16,694
100 nM M100	43815,938	1,2677	43870,4375	4,635445696	78566	1,021213	123873,5	1,6446	1150725	1,4816	2938207	30,615
500 nM M-100	54665,344	1,5816	39664,125	4,190997583	72441	0,9415993	105730,5	1,4037	1684089	2,1683	5042970	52,545
1 µM M-100	61795,938	1,78791	31010,5625	3,276643377	148677	1,9325201	101370,5	1,3459	2219952	2,8582	6426935	66,965
5 µM MS-275	141855,56	4,10423	206463,5	21,81538177	283240	3,6815972	1969546	26,149	1897520	2,4431	742785	7,7394
30 nM LBH589	21490,625	0,62178	310254,6875	32,78218404	25172,5	0,327196	1332087	17,686	261021	0,3361	1842651	19,199

	cl. caspase-3		γH2AX		ac-H3		β-actin	
	Signal	Ratio	Signal	Ratio	Signal	Ratio	Signal	Ratio
Ctl	237449	1	388279,75	1	97540	1	2675506	1
50 nM M-100	207752	0,87493	312471	0,804757396	89549	0,9180746	3273166	1,2234
100 nM M100	234099	0,98589	318152,25	0,819389242	81769	0,8383125	2991326	1,118
500 nM M-100	149853	0,6311	365207,75	0,940578925	81196	0,832438	3679194	1,3751
1 µM M-100	182796	0,76983	882159,5	2,271968858	99656	1,0216937	4032888	1,5073
5 µM MS-275	1310565	5,51935	11250915,38	28,9763125	1209687	12,401958	6388542	2,3878
30 nM LBH589	5654517	23,8136	21267299	54,77313458	749317	7,6821509	5366661	2,0058

HEL - Original blots corresponding to Figure 2A

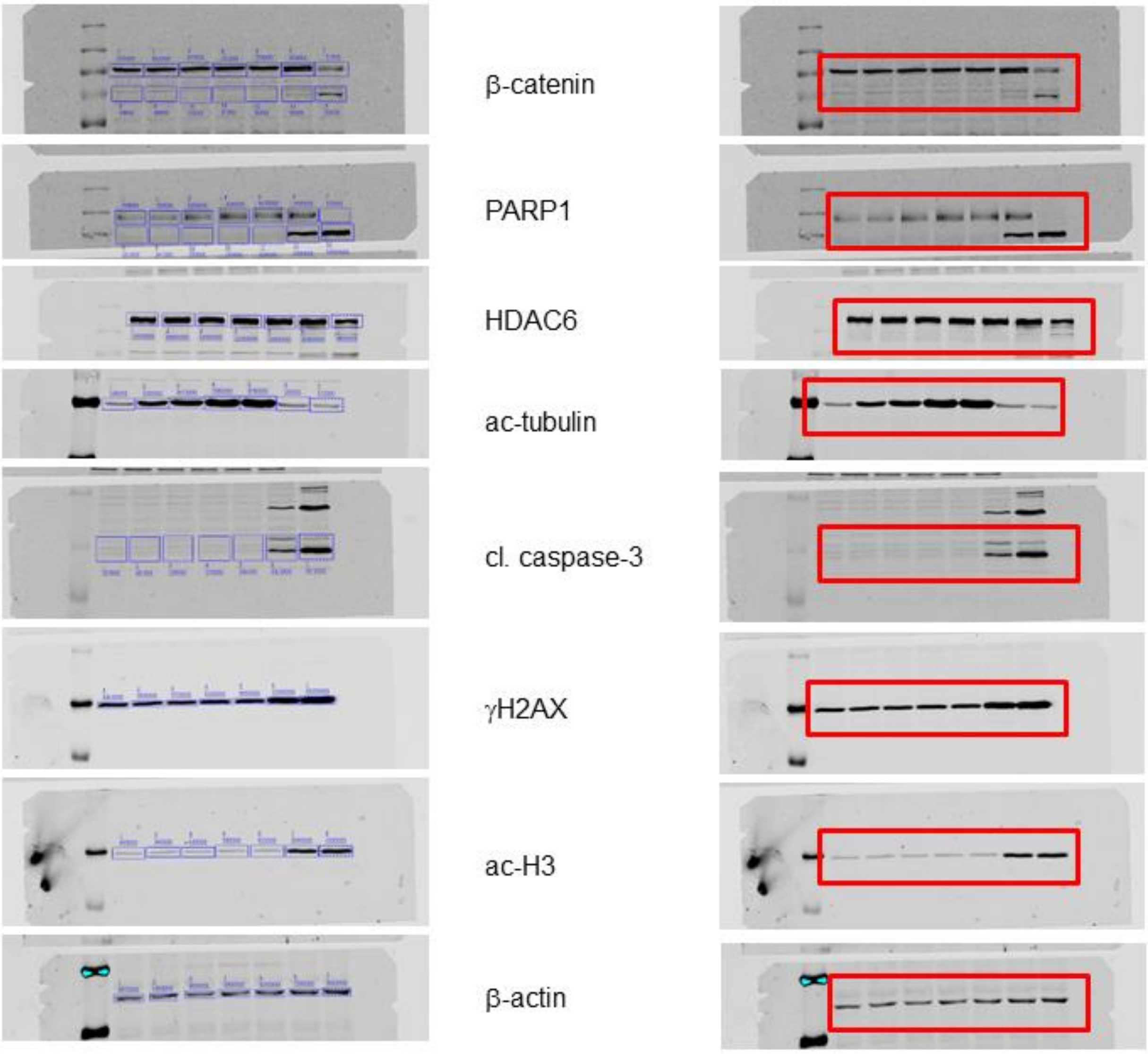

	catenin fl.		catenin cl.		PARP1 fl.		PARP1 cl.		HDAC6		ac-tubulin	
	Signal	Ratio	Signal	Ratio	Signal	Ratio	Signal	Ratio	Signal	Ratio	Signal	Ratio
Ctl	614529	1	54824	1	764438	1	256887	1	14746709	1	240222	1
50 nM M-100	652072	1,06109	48772	0,8896104	780232	1,020661	246660	0,9602	18862501	1,2791	1325469	5,5177
100 nM M100	676511	1,10086	53547	0,9767073	1202925	1,573607	249547	0,9714	19047782	1,2917	3467879	14,436
500 nM M-100	797295	1,29741	57885	1,0558332	1638647	2,143597	210366	0,8189	22464046	1,5233	7681651	31,977
1 µM M-100	770021	1,25303	36891	0,6728987	1419235	1,856573	528467	2,0572	23785521	1,6129	8744212	36,401
5 µM MS-275	608962	0,99094	84258	1,5368817	1657228	2,167904	3363491	13,093	20383458	1,3822	280221	1,1665
30 nM LBH589	170568	0,27756	192357	3,5086276	119560	0,156402	13606352	52,966	8687166	0,5891	171727	0,7149

	cl. caspase-3		γH2AX		ac-H3		β-actin	
	Signal	Ratio	Signal	Ratio	Signal	Ratio	Signal	Ratio
Ctl	296833	1	6344457	1	493362	1	4974126	1
50 nM M-100	281467	0,94823	5816115	0,9167238	546240	1,107179	4857644	0,9766
100 nM M100	237849	0,80129	5720756	0,9016936	482855	0,978703	4693040	0,9435
500 nM M-100	275091	0,92675	6316340	0,9955683	584915	1,18557	5896666	1,1855
1 µM M-100	283590	0,95539	6595413	1,0395552	621180	1,259075	6212540	1,249
5 µM MS-275	1338952	4,51079	22468021	3,5413623	3944581	7,995308	7592024	1,5263
30 nM LBH589	9013231	30,3647	33212211	5,234839	4634662	9,394039	8662796	1,7416

Original blots corresponding to Figure 2B

**ß-catenin**	
Co	71840.382
10 nM	47689.120
30 nM	19842.380
**p-GSK-3**	
Co	33187.103
10 nM	71881.418
30 nM	71852.252
**GSK3**	
Co	84959.373
10 nM	67970.148
30 nM	67692.555
**Ac-tubulin**	
Co	29841.463
10 nM	45438.342
30 nM	74831.361
**PARP fl.**	
Co	27770.836
10 nM	43437.986
30 nM	23460.622
**PARP cl.**	
Co	1227.991
10 nM	14114.572
30 nM	206105.247
**ß-actin**	
Co	27257.706
10 nM	29648.886
30 nM	31958.233

β-catenin

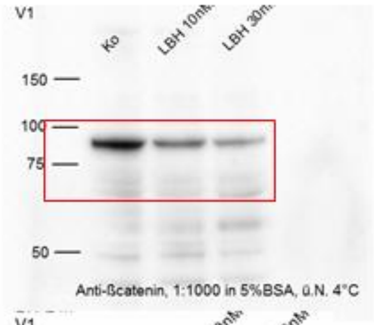

p-GSK3

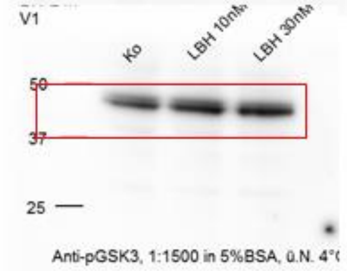

GSK3

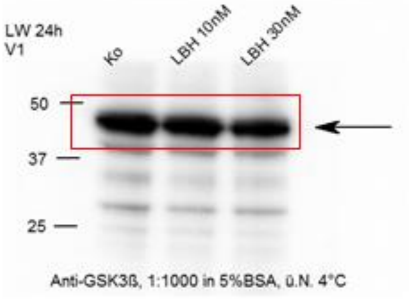

ac-tubulin

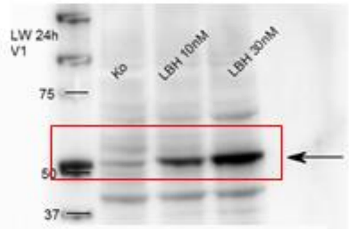

PARP

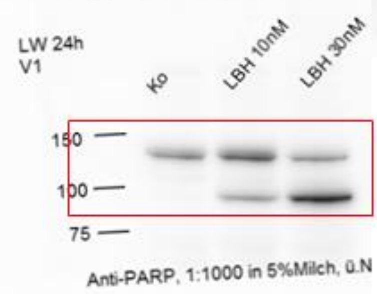

ß-actin

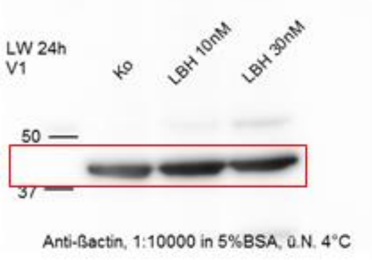

Original blots corresponding to Figure 2C

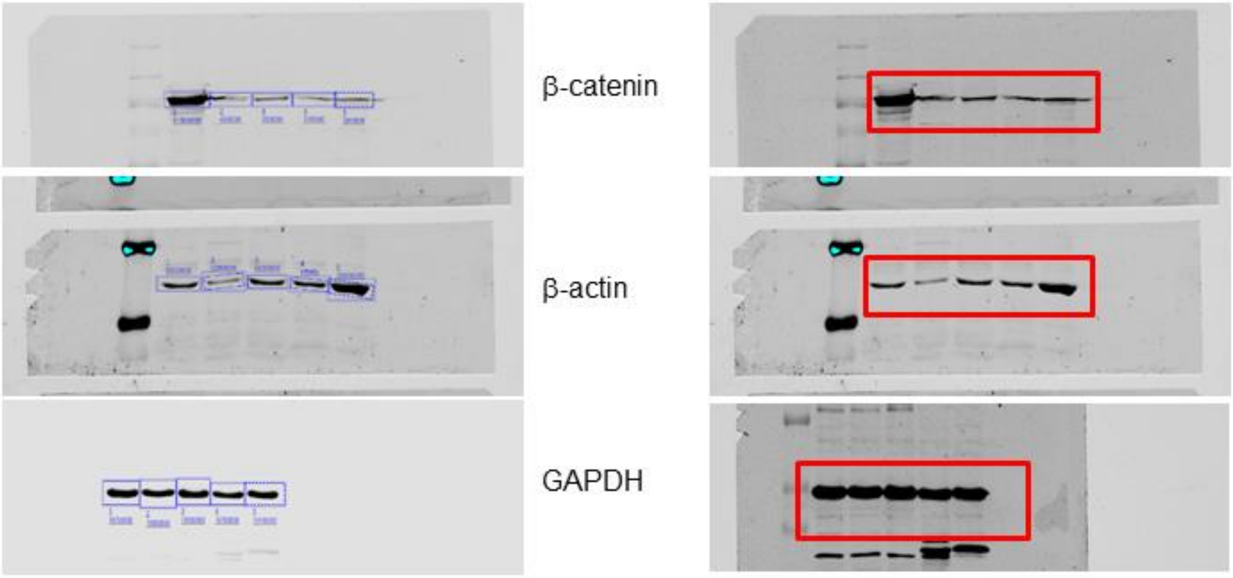

	Signal		
	catenin	actin	GAPDH
HCT116	11456916	4418592	16663006
RKO	406027	1571747	15026161
HEL	293343	4750860	15532152
MV4-11	214606	4137517	10732021
PBMCs	331052	22291575	15076983

Original blots corresponding to Figure 2D

	**ß-catenin**	**p-GSK-3**
Co	6229.790	9819.447
zVAD20	1979.962	10917.811
zVAD50	5438.468	11062.004
10 Pano	4351.690	7522.912
10 Pano 20 zVAD	6261.861	9428.518
10 Pano 50 zVAD	11295.347	74661.467
30 Pano	294.385	7723.154
30 Pano 20 zVAD	6229.790	9514.933
30 Pano 50 zVAD	1979.962	9998.154

	**GSK3**
Co	11362.397
zVAD20	8332.619
zVAD50	9477.669
10 Pano	9158.518
10 Pano 20 zVAD	8891.497
10 Pano 50 zVAD	9462.083
30 Pano	6253.882
30 Pano 20 zVAD	8024.447
30 Pano 50 zVAD	9703.740

	**ß-actin**
Co	9839.664
zVAD20	14655.953
zVAD50	15132.054
10 Pano	14644.518
10 Pano 20 zVAD	15213.983
10 Pano 50 zVAD	13449.589
30 Pano	13826.974
30 Pano 20 zVAD	15893.682
30 Pano 50 zVAD	10150.442

β-catenin

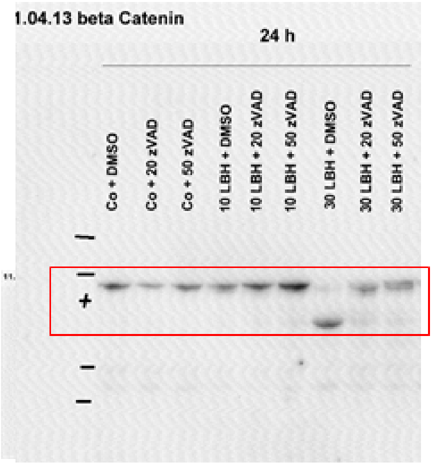

p-GSK3

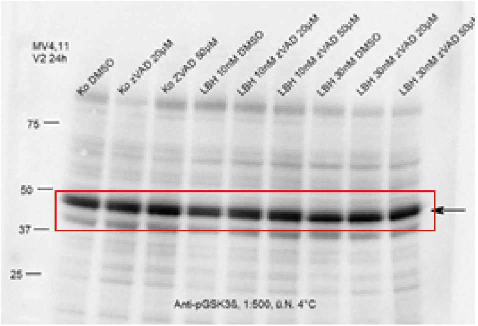

GSK3

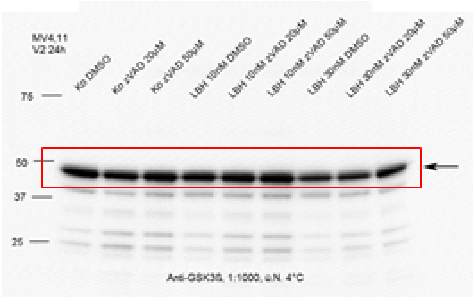

β-actin

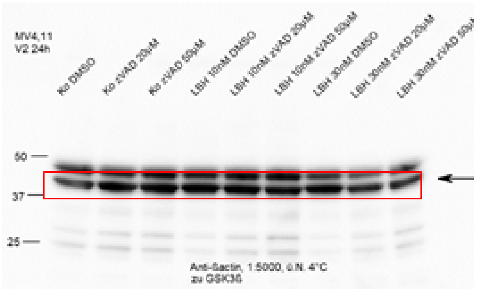

Original blots corresponding to Figure 3A

	ac-H3
Co	1797.719
10 Pano	11886.329
30 Pano	13991.530
50 Pano	21261.522

	PARP fl.	PARP cl.
Co	21703.915	1146.991
10 Pano	6315.664	2646.841
30 Pano	6053.326	4020.276
50 Pano	8272.761	5504.589
	**ß-actin**	
Co	32277.288	
10 Pano	30587.421	
30 Pano	23236.853	
50 Pano	21647.217	

Cut of membrane

ac-H3

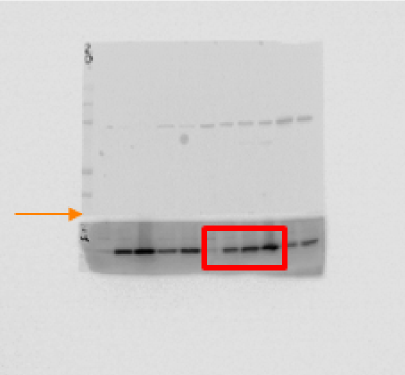

PARP

Cut of membrane

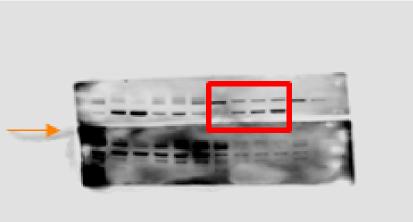

ß-actin

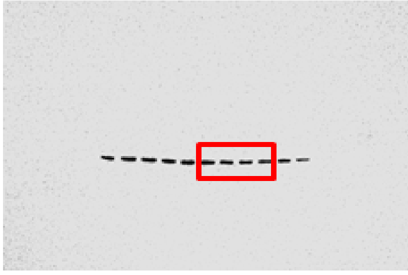

Original blots corresponding to Figure 3B

KG1

	ß-catenin	MYC
Co	6871.844	8077.539
10 Pano	8801.329	3868.468
30 Pano	6623.673	1296.497
50 Pano	7877.572	1064.820

	ac-tubulin	ß-actin
Co	3426.397	14168.631
10 Pano	7828.861	14434.167
30 Pano	9758.690	15839.338
50 Pano	25780.108	13532.451

ß-catenin

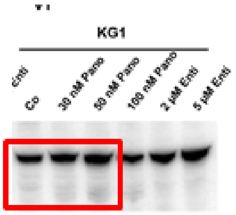

MYC

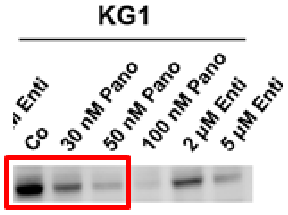

ac-tubulin

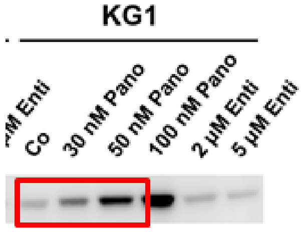

ß-actin

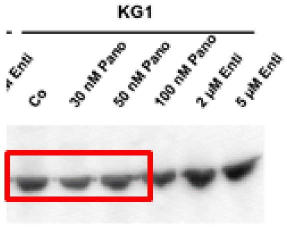

Original blots corresponding to Figure 3B

MV4-11

ß-catenin fl.	
Co	5510.761
10 Pano	7355.995
30 Pano	1910.276

β-catenin cl.

Co	627.335
10 Pano	630.062
30 Pano	6982.045
**MYC**	
Co	33777.945
10 Pano	4102.640
30 Pano	1898.577
**ac-tubulin**	
Co	1399.184
10 Pano	2501.790
30 Pano	5412.610
**ß-actin**	
Co	10757.489
10 Pano	7055.238
30 Pano	5165.066

ß-catenin

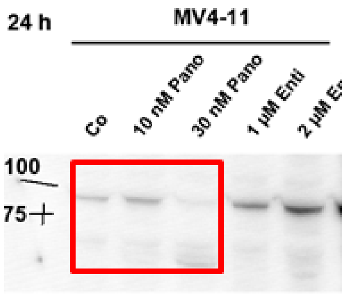

MYC

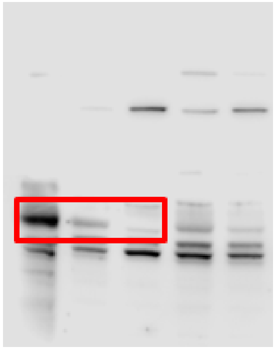

ac-tubulin

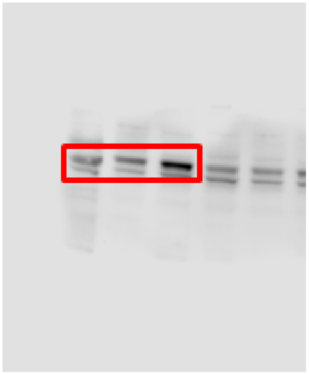

ß-actin

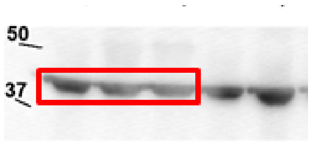

Original blots corresponding to Figure 3C

ß-catenin	
Co	11244.983
zVAD20	11420.619
zVAD50	10956.983
10 Pano	10481.326
10 Pano 20 zVAD	10482.983
10 Pano 50 zVAD	12456.104
30 Pano	10464.933
30 Pano 20 zVAD	8796.740
30 Pano 50 zVAD	8654.740

PARP upper	
Co	11432.518
zVAD20	9539.205
zVAD50	10246.205
10 Pano	12161.033
10 Pano 20 zVAD	11538.518
10 Pano 50 zVAD	5015.276
30 Pano	5592.205
30 Pano 20 zVAD	3555.912
30 Pano 50 zVAD	3835.841

PARP lower	
Co	3190.669
zVAD20	370.749
zVAD50	25151.120
10 Pano	13289.690
10 Pano 20 zVAD	882.841
10 Pano 50 zVAD	1883.497
30 Pano	683.941
30 Pano 20 zVAD	14461.740
30 Pano 50 zVAD	1900.548

c-myc	
Co	5934.740
zVAD20	11004.004
zVAD50	11350.276
10 Pano	8771.740
10 Pano 20 zVAD	10138.619
10 Pano 50 zVAD	11109.983
30 Pano	5193.882
30 Pano 20 zVAD	2634.912
30 Pano 50 zVAD	3007.790

β-catenin

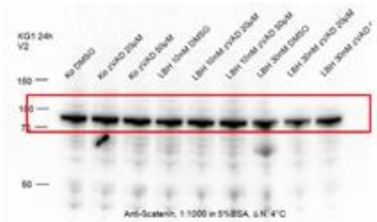

PARP

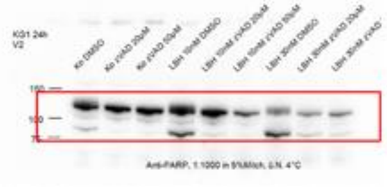

c-myc

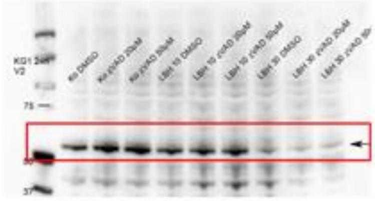

β-actin

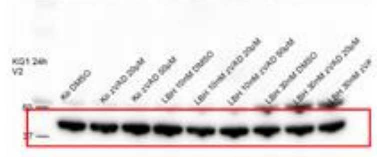

ß-actin 1	
Co	11270.125
zVAD20	9056.983
zVAD50	10238.711
10 Pano	11061.439
10 Pano 20 zVAD	8887.518
10 Pano 50 zVAD	11277.983
30 Pano	10601.861
30 Pano 20 zVAD	10423.690
30 Pano 50 zVAD	11207.388

Original blots corresponding to Figure 3C continued

p-GSK3	
Co	7275.861
zVAD20	7683.083
zVAD50	6846.669
10 Pano	7941.326
10 Pano 20 zVAD	7834.497
10 Pano 50 zVAD	7570.912
30 Pano	8343.619
30 Pano 20 zVAD	10394.983
30 Pano 50 zVAD	11938.690

phospho-GSK3

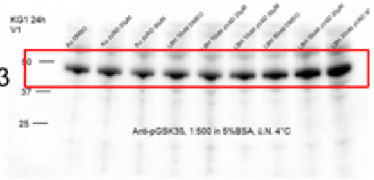

GSK3	
Co	10379.983
zVAD20	9939.205
zVAD50	11139.154
10 Pano	12351.983
10 Pano 20 zVAD	12320.276
10 Pano 50 zVAD	10914.790
30 Pano	11405.740
30 Pano 20 zVAD	10266.912
30 Pano 50 zVAD	12228.882

GSK3

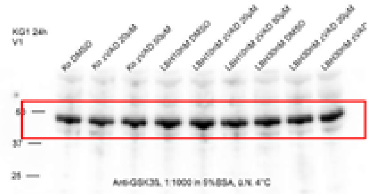

ß-actin 2	
Co	18897.823
zVAD20	24012.087
zVAD50	28241.451
10 Pano	24869.602
10 Pano 20 zVAD	34791.229
10 Pano 50 zVAD	35149.907
30 Pano	27303.693
30 Pano 20 zVAD	39396.877
30 Pano 50 zVAD	39689.635

β-actin

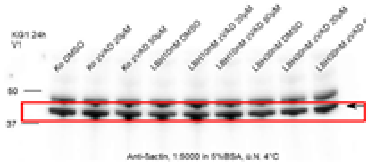

Original blots corresponding to Figure 3D

	**ß-catenin**	**ß-actin**
Co	43311.061	10817.761
Indo	34211.957	10051.953

β-catenin

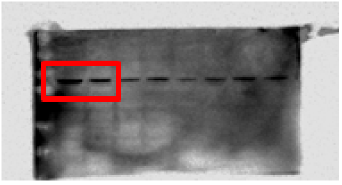

β-actin

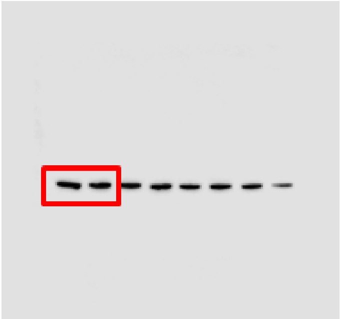

Original blots corresponding to Figure 4C

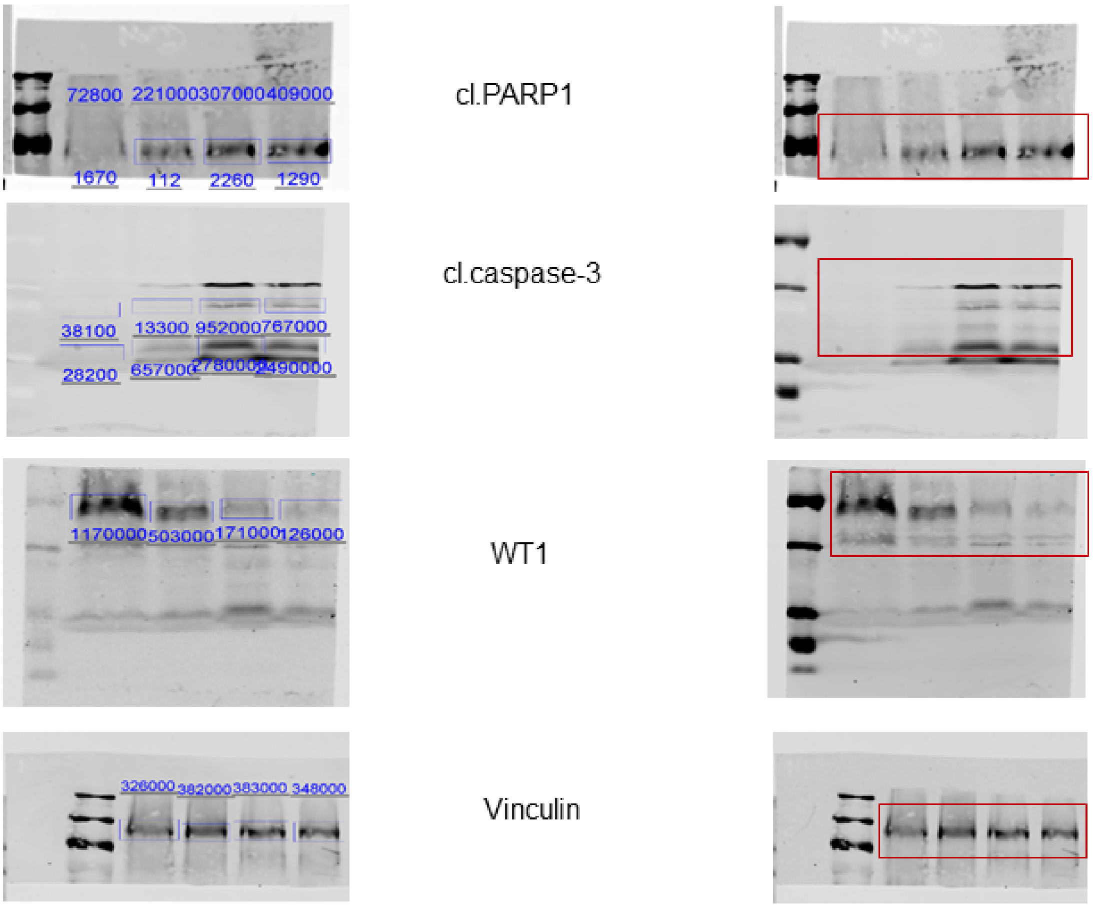

MV4-11	Vinculin		PARP1. cl		cl. caspase-3 (i)		cl. caspase-3 (ii)		WT1	
	Signal	Ratio	Signal	Ratio	Signal	Ratio	Signal	Ratio	Signal	Ratio
10 µM RGFP966	348000	1.0674847	409000	1.2546012	767000	2.3527607	2490000	7.6380368	126000	0.3865031
5 µM RGFP966	383000	1.1748466	307000	0.9417178	952000	2.9202454	2780000	8.5276074	171000	0.5245399
1 µM RGFP966	382000	1.1717791	221000	0.6779141	13300	0.0407975	757000	2.3220859	503000	1.5429448
Ctl.	326000	1	72800	0.2233129	38100	0.1168712	28200	0.0865031	1170000	3.5889571

Original blots corresponding to Figure 4D

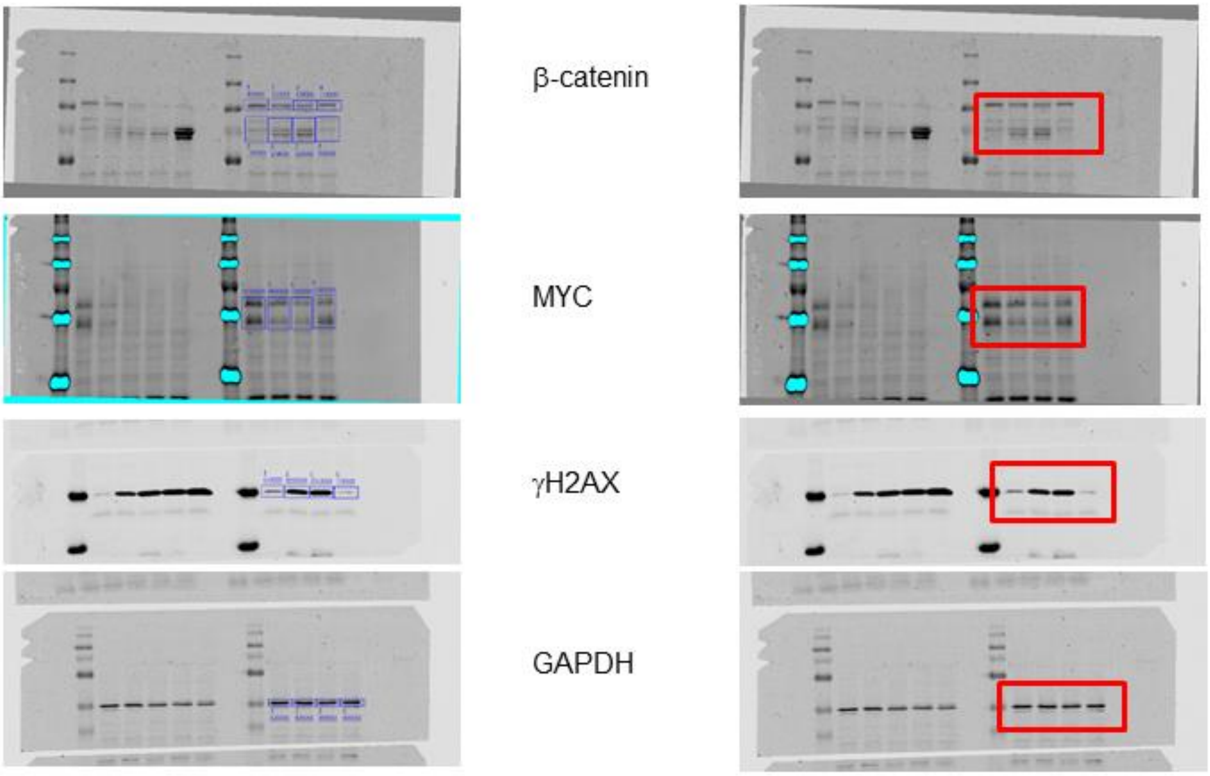

	GAPDH		catenin fl.		catenin cl.		MYC		γH2AX	
	Signal	Ratio	Signal	Ratio	Signal	Ratio	Signal	Ratio	Signal	Ratio
1 µM RGFP966	426176	1,06502	158929,25	1,349584383	190303	1,4899316	19149080	1,6029	442732	2,5168
5 µM RGFP966	430120	1,07488	121799	1,034284301	274368	2,1480973	9418000	0,7884	2997564	17,04
10 µM RGFP966	358697	0,89639	107528	0,913098813	328427	2,5713392	7299288	0,611	3739094	21,256
Ctl	400156	1	117761,625	1	127726	1	11946360	1	175910	1

Original blots corresponding to Figure 5A

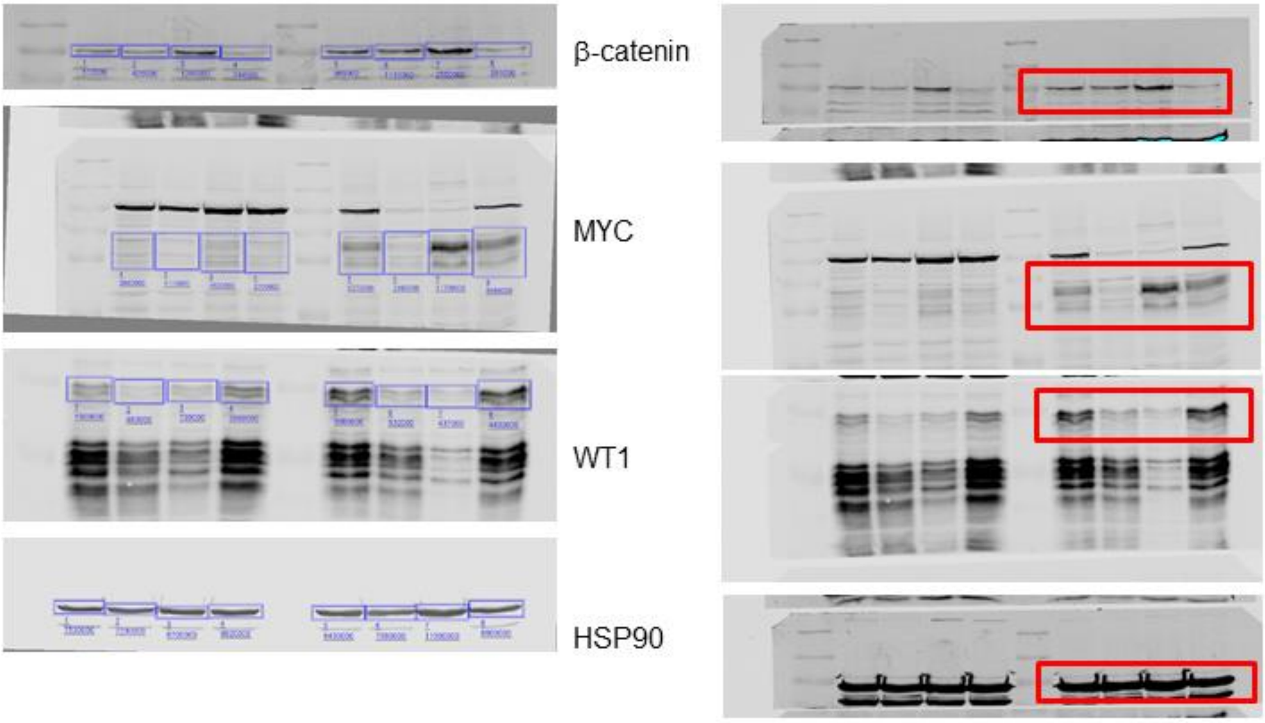

	HSP90		MYC		WT1		catenin	
	Signal	Ratio	Signal	Ratio	Signal	Ratio	Signal	Ratio
1 NC siRNA MV (II)	8431771	1	6272130,25	1	3081069	1	989258	1
2 siMYC (II)	7591129,5	0,9003	2162387	0,344761176	931521	0,302337	1105596	1,1176
3 siWT1 (II)	11288387	1,33879	11690633,75	1,863901623	437344	0,141946	2551302	2,579
3 siβ-catenin (II)	8795778,8	1,04317	8963941,75	1,429170217	4429075	1,437512	381148,5	0,3853

Original blots corresponding to Supplement 3

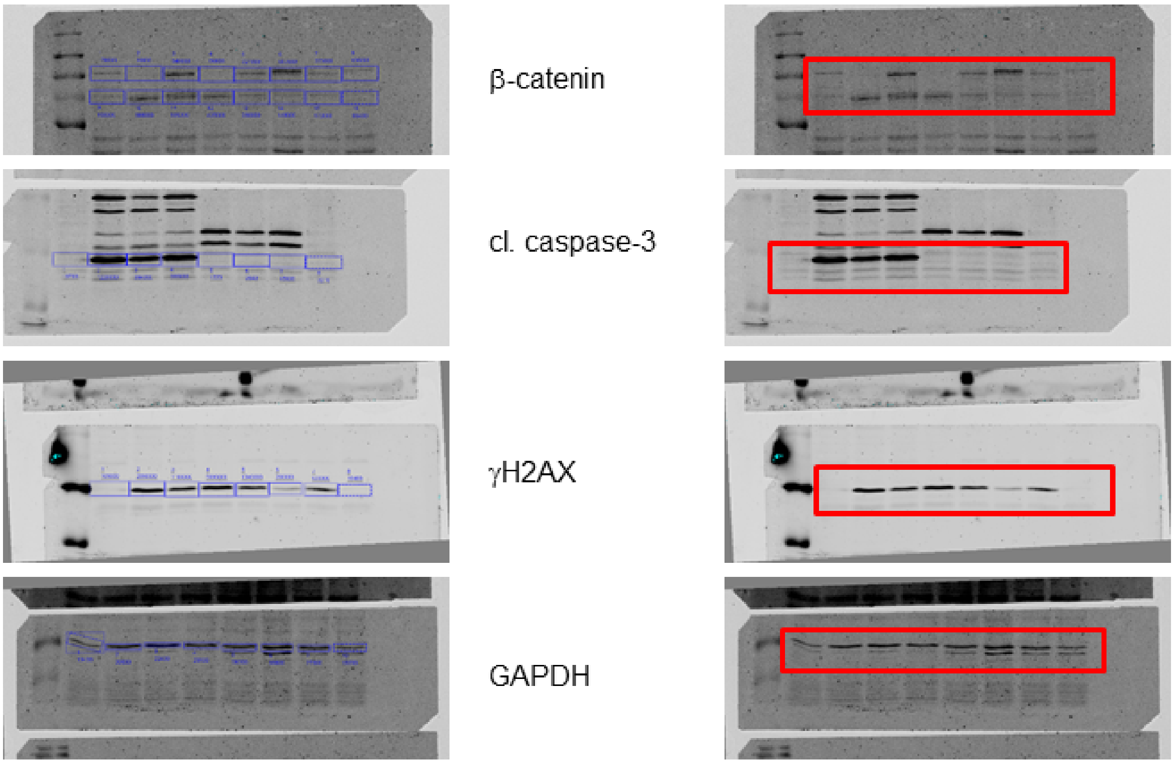

	catenin fl.		catenin cl.		cl. caspase-3		γH2AX		GAPDH	
	Signal	Ratio	Signal	Ratio	Signal	Ratio	Signal	Ratio	Signal	Ratio
Ctl	177794	1	166475	1	19654	1	169208,8	1	19352	1
30 nM LBH589	93838	0,52779	387820	2,329599	1224952	62,32584	2894281	17,105	30777	1,5904
5 µM MS-275	547944	3,0819	506464	3,0422826	384306	19,55358	1177059	6,9563	22418	1,1585
10 nM FK228	56772	0,31931	424804	2,5517585	935233	47,58487	2086148	12,329	28100	1,4521
30 nM LBH589 + 40 µM z-VAD	320993	1,80542	230342	1,3836432	1221	0,062125	1038239	6,1358	28207	1,4576
5 µM MS-275 + 40 µM z-VAD	687210	3,8652	148318	0,8909326	3841	0,195431	290239,3	1,7153	33769	1,745
10 nM FK228 + 40 µM z-VAD	222382	1,25078	172272	1,034822	15578	0,792612	933287	5,5156	27280	1,4097
40 µM z-VAD	135819	0,76391	80429	0,4831296	3210	0,163326	19385,75	0,1146	19237	0,9941

Original blots corresponding to Supplement 5

	**MYC**
Co	80696.122
10 nM	53472.328
30 nM	18146.966

	**ac-H3**
Co	19492.187
10 nM	64676.943
30 nM	89869.820

	**β-actin**
Co	82810.696
10 nM	61016.236
30 nM	77822.671

c-myc

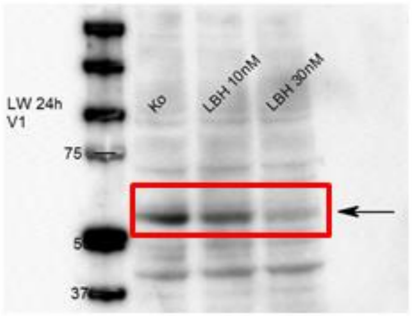

ac-H3

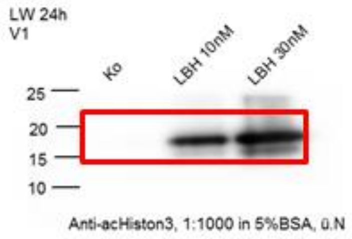

β-actin

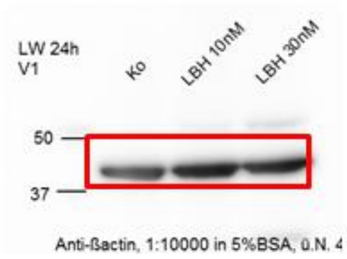

Original blots corresponding to Supplement 7

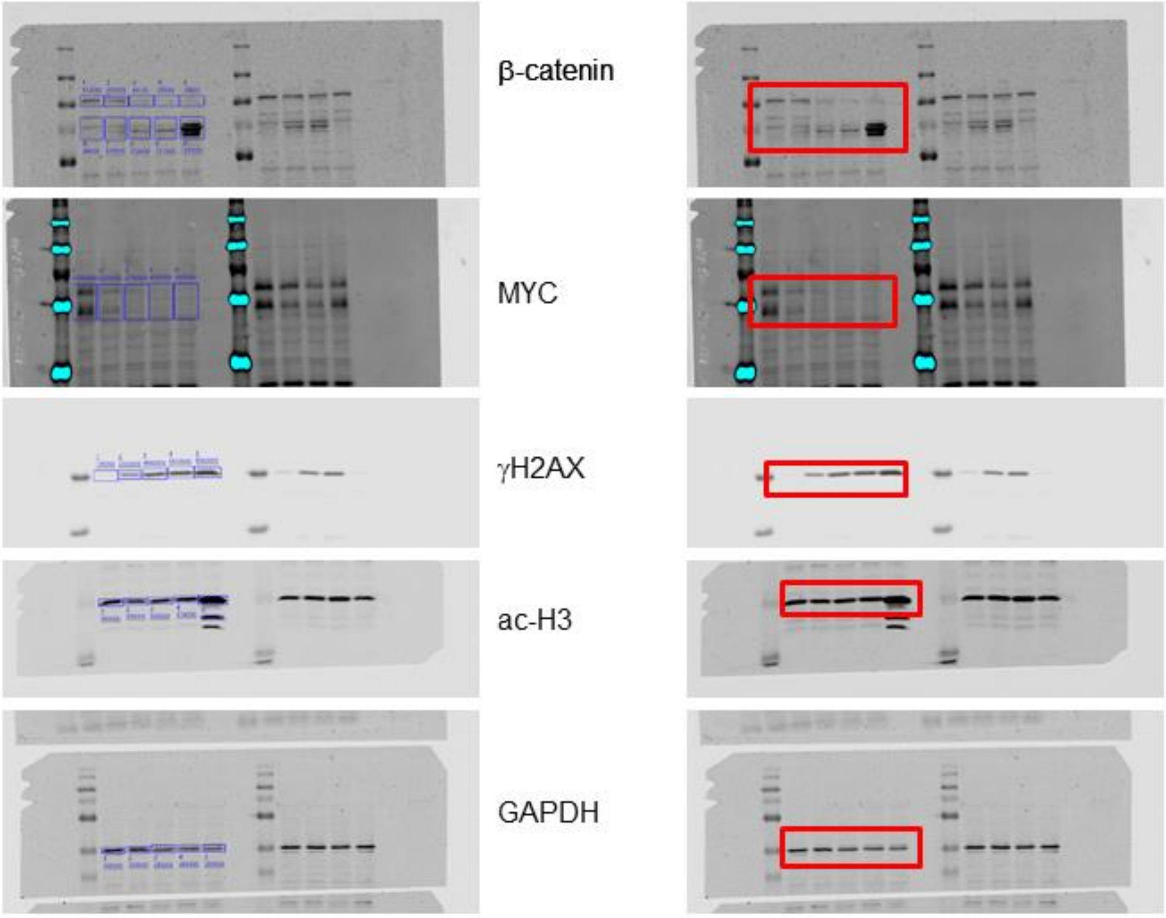

	catenin fl.		catenin cl.		MYC		γH2AX		ac-H3		GAPDH	
	Signal	Ratio	Signal	Ratio	Signal	Ratio	Signal	Ratio	Signal	Ratio	Signal	Ratio
Ctl	112173	1	86583	1	15321924	1	134827	1	593301	1	335295	1
0.5 mM HU	100356	0,89465	127333	1,4706467	6262684	0,40874	2324132	17,238	399061	0,6726	332616	0,992
1.5 mM HU	45691	0,40733	151553	1,7503782	4725680	0,308426	4660211	34,564	505804	0,8525	291618	0,8697
5 mM HU	28974	0,2583	150984	1,7438065	4164024	0,271769	5611731	41,622	673941	1,1359	265081	0,7906
30 nM LBH589	38563	0,34378	1415889	16,352968	3018716	0,197019	8363724	62,033	4357711	7,3449	255276	0,7613
